# Supplementary material for: Predicting visual function by interpreting a neuronal wiring diagram
Source: Nature. 2024 Oct 2;634(8032):113–23. doi: 10.1038/s41586-024-07953-5 (PMC11446822; doi:10.1038/s41586-024-07953-5)
Supplement: Supplementary file 6 — CRF and ERF predictions for individual TmY4 and TmY9 cells. Analogous to Supplementary Data 3, but for TmY target types. Shown are the top four monosynaptic pathways, the strongest pathway passing through each of the top ten intermediary types (ranking from Extended Data Fig. 7), and the trisynaptic pathway Tm1–TmY–Dm3–TmY (see the section entitled Prediction of spatial normalization). [file 41586_2024_7953_MOESM6_ESM.zip › DataS4/TmY9qΓèÑ/720575940638416361.pdf]

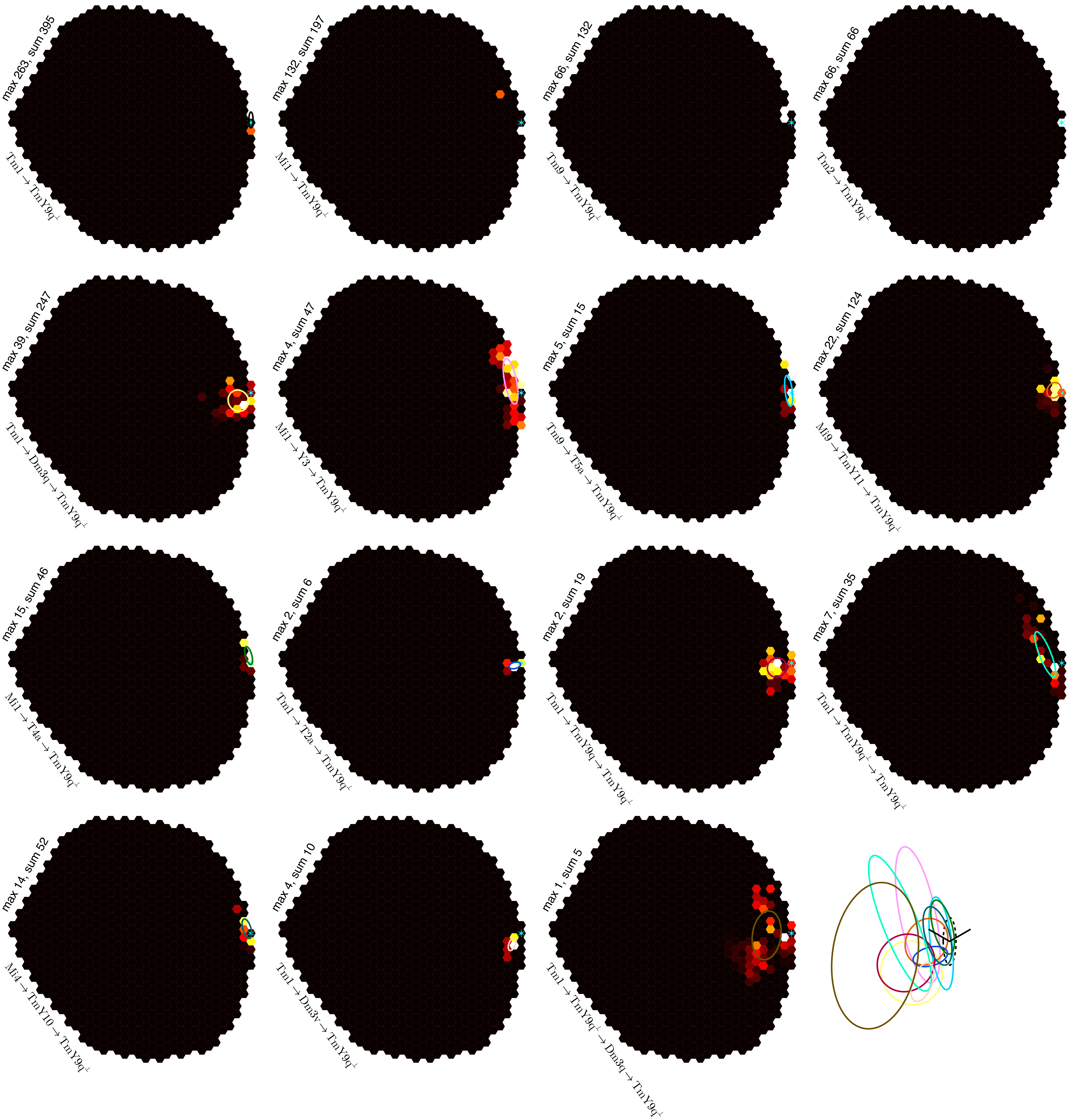
max 263, sum 395
Tm1 → TmY9q$^{\perp}$
max 132, sum 197
Mi1 → TmY9q$^{\perp}$
max 66, sum 132
Tm9 → TmY9q$^{\perp}$
max 66, sum 66
Tm2 → TmY9q$^{\perp}$
max 39, sum 247
Tm1 → Dm3q → TmY9q$^{\perp}$
max 4, sum 47
Mi1 → Y3 → TmY9q$^{\perp}$
max 5, sum 15
Tm9 → T5a → TmY9q$^{\perp}$
max 22, sum 124
Mi9 → TmY11 → TmY9q$^{\perp}$
max 15, sum 46
Mi1 → T4a → TmY9q$^{\perp}$
max 2, sum 6
Tm1 → T2a → TmY9q$^{\perp}$
max 2, sum 19
Tm1 → TmY9q → TmY9q$^{\perp}$
max 7, sum 35
Tm1 → TmY9q$^{\perp}$ → TmY9q$^{\perp}$
max 14, sum 52
Mi4 → TmY10 → TmY9q$^{\perp}$
max 4, sum 10
Tm1 → Dm3v → TmY9q$^{\perp}$
max 1, sum 5
Tm1 → TmY9q$^{\perp}$ → Dm3q → TmY9q$^{\perp}$
